# Family events and child behavior in late childhood: a cross-sectional study

**DOI:** 10.1186/s12887-024-05233-9

**Published:** 2024-11-20

**Authors:** Rikuya Hosokawa, Riho Tomozawa, Megumi Fujimoto, Toshiki Katsura

**Affiliations:** 1 Department of Human Health Sciences, Graduate School of Medicine, Kyoto University, 53 Shogoin-kawahara-cho, Sakyo-ku, Kyoto, 606-8507 Japan; 2 Faculty of Nursing Science, Osaka Seikei University, Osaka, 533-0007 Japan; 3 Faculty of Nursing, Meiji University of Integrative Medicine, Kyoto, 629-0392 Japan

**Keywords:** Family events, Problematic behavior, Prosociality, Late childhood, Social adjustment

## Abstract

**Background:**

Children’s social behavior develops through diverse experiences. However, the relationship between children’s experiences of celebratory events and their behavioral development has not been previously quantified. Therefore, this study aimed to quantitatively explore this relationship.

**Methods:**

In 2020, a self-reported questionnaire was completed by 653 sixth-grade students (aged 11–12 years) and/or their caregivers in Nagoya, Aichi, a major metropolitan area in Japan. The main items surveyed were children’s experiences with events celebrated by their family and their behavioral development. The Strengths and Difficulties Questionnaire was used to assess children’s behavior. This questionnaire identifies behavioral and emotional problems and prosocial behavior. Multiple regression analysis was conducted with the number of family events experienced by children as the explanatory variable and their emotional and prosocial behavior as the objective variables.

**Results:**

Children from families who participated in fewer family events tended to have higher problematic behavior risk scores and lower prosociality scores compared with their counterparts. Compared with children who participated in more than 20 family events, those who participated in fewer than 10 family events had more than three times the risk for exhibiting problem behaviors (odds: 3.558, 95% CI: 1.252–10.111, *p* = .017) and prosocial problems (odds: 3.184, 95% CI: 1.726–5.876, *p* < .001). Conversely, children from families who participated in more family events tended to have lower problematic behavior risk scores and higher prosociality scores.

**Conclusions:**

A higher number of family events may reduce the risk of behavioral problems in children and increase their prosociality. Enjoying family events may be beneficial for social adjustment.

**Supplementary Information:**

The online version contains supplementary material available at 10.1186/s12887-024-05233-9.

## Background

We often encounter various forms of domestic events in our daily lives, including family events that are universal to human culture and embedded within the cultural and ecological context of family life [1]. These events are uniquely meaningful family interactions that express a family’s beliefs and values and provide individuals with a sense of stability and identity, as well as a means of socialization [2]. Although the specifics of ritual events vary between cultures, all cultures have events with contextual demands [1]. Family events are common in everyday life and widespread across cultures. Owing to globalization, they have become increasingly diverse in many countries, including Japan, and now include a mixture of traditional annual and other events adopted from foreign cultures.

Events have a social function, and many ritual acts derive their meaning from the conventionality of the ritual rather than from the consequences of the act [3]. This meaning is transmitted across social groups through imitation, a powerful learning mechanism used by children to acquire information [4]. Children learn beneficial social conventions through mimicry, especially by imitating their parents [5–7]. Evidence suggests that children perceive ritualized behaviors as socially beneficial and normative [8]. Events are not only enjoyable but also significant in the acquisition of cultural knowledge and in teaching children how to navigate the physical and social worlds, often without being aware of their dependence on that knowledge. This knowledge includes understanding certain ritualized artifacts, such as gifts and attire. There is evidence that an understanding of events emerges early in life and is built on social concepts and idiosyncratic processes [9]. Participating in events helps form, maintain, and promote cooperation within a family.

Furthermore, simply observing events can influence a child’s identity, religious beliefs, memories, and emotions. Overall, a child’s sensitivity to ritual cues can affect the fidelity of their imitation or the degree to which they bond within a group. Regarding participation in events, we assumed that upper elementary school students might be more spontaneous and might actively participate in events with their families. Therefore, in this study, we targeted upper elementary school students because lower elementary school students might be passive, while middle school students and older might spend more time outside the home with friends and other family members.

Events have varied effects [10–12]. Annual family events have the potential to promote children’s healthy development. Children grow by observing adult behavior and incorporating what they learn into their lives. Additionally, they can learn about the seasons through annual events. Japan has four seasons; the colorful indoor decorations for each event, the taste and texture of traditional dishes, and the hot or cold weather experienced during these events stimulate children’s senses and nurture their curiosity.

### Current study

Children’s social behavior develops through a variety of experiences, but the relationship between family event experiences and behavioral development has not been quantitatively clarified. Therefore, this study aimed to investigate whether there is a relationship between the diversity of experiences of intrafamily events and children’s behavior and quantitatively clarify this aspect. We hypothesized that children with fewer experiences of intrafamily events would be at a higher risk of problem behavior. If evidence shows that enjoying seasonal events as a family promotes children’s development and decreases problematic behavior, the risk of problematic behavior may decrease.

## Methods

### Participants

In 2014, we recruited parents and children from 80 kindergartens and daycare centers in Nagoya, one of the most urban areas in Japan. Of the 5024 families invited to participate in the study, 3314 agreed. We have conducted surveys annually since 2014, and a 10-year follow-up survey has also been planned. The current study is part of a more extensive cohort study and uses data from 2020. Owing to some participants relocating or otherwise not being tracked, the questionnaires were distributed to 1345 families in 2020, of whom 707 (response rate: 52.6%) responded. The questionnaire used in our study was developed specifically for this research. After excluding the responses of children with developmental disabilities (*n* = 54), 653 children’s responses were included in the analysis, and the relationship between the number of experiences that children have while celebrating family events and children’s behavior was examined.

A self-reported questionnaire was completed by 653 sixth-grade students (aged 11–12 years) and/or their caregivers. The participant characteristics are presented in Table 1. The mean age of the parents was 44.05 (± 4.43) years for mothers and 45.92 (± 5.42) years for fathers. The most common educational level of the parents was junior college or vocational school for 270 (41.35%) of the mothers and university or graduate school for 388 (59.42%) of the fathers. The parents’ income ranged from 4 to 8 million JPY, most frequently for 364 (55.74%) of the parents. The family structures of the sample were single parent: 43 (6.58%) and two parents: 610 (93.42%). The average age of the children was 12.14 (± 0.35) years, with 312 (47.78%) boys and 341 (52.22%) girls.

**Table 1:** Target attributes

Variable	*n*	%
Child’s sex		
Boy	312	47.78
Girl	341	52.22
Family composition		
Single-parent family	43	6.58
Two-parent family	610	93.42
Number of siblings		
0	100	15.31
≥ 1	553	84.69
Annual household income (million JPY)		
< 4	112	17.15
4–8	364	55.74
≥ 8	165	25.27
Maternal educational level		
Middle school or high school	128	19.60
Junior college or vocational school	270	41.35
University or graduate school	249	38.13
Paternal educational level		
Middle school or high school	154	23.58
Junior college or vocational school	90	13.78
University or graduate school	388	59.42
Total		
-	653	100.00

### Measurement

#### Objective variable: behavior

Behavior was assessed using the Strengths and Difficulties Questionnaire (SDQ) [13–15]. The SDQ is a short questionnaire in which parents or school teachers answer 25 questions about their child’s/student’s emotions and behavior. Dr. Robert Goodman developed the SDQ in the United Kingdom as a screening instrument for children’s overall mental health. It has been tested for reliability and validity and is now used worldwide for screening, clinical assessment, and various research purposes. It is a widely validated brief screening instrument for identifying behavioral and emotional problems and prosocial behavior. This study used the highly reliable and valid Japanese version of the SDQ [16]. The SDQ assesses behavioral and emotional problems and prosocial behavior on a 3-point Likert scale (0‒2 points). Emotional and behavioral problems include conduct problems, hyperactivity and inattention, emotional symptoms, and issues with peers, with higher scores indicating greater emotional and behavioral problems. Similarly, higher scores for prosociality indicate stronger prosociality. Regarding the cutoff values for total difficulties, a score of 16 or higher is considered abnormal, whereas for prosociality, a score of 4 or lower is considered abnormal [16]. Formal permission to use the SDQ in this study was obtained from Youth in Mind, authorizing its inclusion in our publication.

### Explanatory variable: number of diverse annual family events

The participants were asked whether the children celebrated 34 major events with family in Japan, including seasonal events held during spring, summer, fall, and winter, and celebratory events, including birthdays and wedding anniversaries. The operational definition of this variable was based on whether or not these events were held, with the total number of events defined as the number of events per year. The number of annual events was ascertained through a questionnaire survey of the parents.

### Adjustment variables

The participants were asked about their sex, family structure, presence of siblings, annual household income, and parents’ educational levels.

### Main statistical analysis

The quantitative association between family events and children’s behavior was investigated by conducting logistic regression analysis. The first model was a crude design with no adjustment variables. Adjustment variables (child’s sex, family composition, presence of siblings, annual household income, maternal educational level, and paternal educational level) were included in the second model, making it an adjusted model.

The association between the participant demographics and the number of annual family events attended was investigated by conducting a chi-square test. Independent t-tests and logistic regression analysis were conducted to examine the association between the number of family events and children’s problematic behaviors and prosociality. All analyses were conducted using IBM SPSS Statistics software version 29.

## Results

### Family events

The list of events used in the survey and the number of events celebrated by the participants are presented in Table 2. Examples of events include children’s birthdays, Christmas (events: decorating the Christmas tree, eating Christmas cake, etc.), New Year’s (events: Hatsumode, eating New Year’s dishes, etc.), New Year’s Eve (events: eating soba noodles before midnight, etc.), Valentine’s Day (events: giving and receiving chocolates, etc.), siblings’ birthdays, and Setsubun (events: bean-throwing, Ehomaki, etc.). The execution of traditional Japanese events that inspire happiness exceeded 80%. Table 3 presents the number of annual family events attended by each family.

**Table 2:** List of ritual events

Month	Content	*n*	%
Jan	New Year (e.g., shrine visit, eating traditional dishes, etc.)	625	95.71
Jan	Nanakusa (eating seven spring plant soup on the seventh day of the New Year)	202	30.93
Jan	Kagami-biraki (cracking a huge rice cake and eating it with soup)	256	39.20
Feb	Setsubun (bean-throwing, eating Ehomaki sushi, etc.)	530	81.16
Feb	Valentine’s Day (giving and/or receiving chocolates)	558	85.45
March	Doll’s Festival (displaying Hina dolls and eating special rice crackers and tossed sushi)	450	68.90
March	White Day (giving and/or receiving presents)	331	50.69
March	Spring Equinoctial week (grave visit, eating special rice cakes)	177	27.11
April	Easter (eating egg dishes, special feasts, etc.)	38	5.82
April	Viewing cherry blossoms (eating rice cakes in cherry leaves)	382	58.50
April	Children’s Day (displaying carp streamers, eating special sweets, etc.)	488	74.70
May	Mother’s Day (gifts to mothers)	408	62.48
June	Father’s Day (gifts to fathers)	384	58.80
July	Tanabata (Weaver’s Festival, bamboo decorations, etc.)	215	32.92
Aug	Bon Festival (grave visit)	436	66.77
Aug	Midsummer Day of the Ox (eating grilled eel)	273	41.81
Sept	Autumn Equinoctial week (grave visit, eating special rice cakes)	186	28.48
Sept	Moon viewing (eating special dumplings)	246	37.67
Sept	Respect for the Aged Day (gifts to grandparents)	271	41.50
Oct	Halloween (costumes and decorations)	317	48.55
Nov	Viewing of autumn leaves	178	27.26
Dec	Winter solstice (taking a hot bath, eating pumpkin dishes, etc.)	306	46.86
Dec	Christmas (tree decoration, eating cake)	629	96.32
Dec	New Year’s Eve (eating traditional soba noodles)	594	90.96
-	Children’s birthdays	643	98.47
-	Siblings’ birthdays	543	83.15
-	Parents’ birthdays	476	72.89
-	Grandparents’ birthdays	294	45.02
-	Birthdays of cousins and other relatives	101	15.47
-	Parents’ wedding anniversary	147	22.51
-	Picking seasonal foods (strawberries, grapes, bamboo shoots, etc.)	200	30.63
-	Going to see seasonal insects and animals (fireflies, migratory birds, etc.)	101	15.47
-	Local festivals	375	57.43
-	Others	29	4.44

**Table 3:** Number of annual family events attended

No. events	*n*	%
≥ 21	96	14.70
16–20	150	22.97
11–15	196	30.02
0–10	211	32.31

### Participant demographics and number of annual family events celebrated

The participants’ demographics and the number of annual family events celebrated are presented in Table 4. Girls participated in more activities than boys, two-parent families participated in more events than single-parent families, and children with siblings participated in more events than those without siblings.

**Table 4:** Target attributes and number of annual family events

Variable	M	SD	*P*
Child’s sex			
Boy	16.48	6.15	< 0.001
Girl	18.32	5.99	
Family composition			
Single-parent family	14.35	7.59	< 0.001
Two-parent family	17.66	5.97	
Number of siblings			
0	15.50	6.63	< 0.001
≥ 1	17.79	5.98	
Annual household income (million JPY)			
< 4	16.54	6.57	0.281
4–7	17.45	6.26	
≥ 8	17.57	5.85	
Maternal educational level			
Middle school or high school	16.41	6.40	0.078
Junior college or vocational school	17.57	6.12	
University or graduate school	17.88	5.84	
Paternal educational level			
Middle school or high school	17.47	6.29	0.987
Junior college or vocational school	17.49	6.46	
University or graduate school	17.56	5.87	
Total			
-	17.44	6.14	-

Note: P-values are calculated by conducting a t-test and a one-way analysis of variance (ANOVA)

### Association between the number of annual family events and children’s behavior

Table 5 presents the association between the number of family events and children’s problematic behaviors, and Table 6 presents the association between the number of family events and children’s prosociality.

**Table 5:** Number of seasonal events and children’s problem behavior scores

No. events	M	SD	*P*
≥ 21	6.80	4.25	0.007
16–20	7.20	4.95	
11–15	7.70	4.90	
0–10	8.82	5.19	

Note: P-values are calculated by conducting a one-way analysis of variance (ANOVA)

**Table 6:** Number of seasonal events and children’s prosociality scores

No. events	M	SD	*P*
≥ 21	6.97	2.10	< 0.001
16–20	6.51	2.19	
11–15	6.12	2.25	
0–10	5.87	2.06	

Note: P-values are calculated by conducting a one-way analysis of variance (ANOVA)

The results demonstrate that children from families who participated in fewer family events tended to have higher risk scores for problematic behaviors and lower scores for prosociality. In the adjusted model, children who attended 0–10 family events had more than three times the risk of exhibiting problem behaviors (odds: 3.558, 95% CI: 1.252–10.111, *p* = .017) and abnormal prosociality (odds: 3.184, 95% CI: 1.726–5.876, *p* < .001) than children who attended ≥ 21 family events (Tables 7 and 8). Furthermore, the same results were obtained in the multiple regression analysis (Supplementary Table-1 and Supplementary Table-2).

**Table 7:** Relationship between the number of seasonal events and children’s problem behaviors

No. events	Crude model				Adjusted model			
	Odds	95% CI		*P*	Odds	95% CI		*P*
≥ 21	Ref				Ref			
16–20	1.850	0.659	5.191	0.243	1.692	0.576	4.969	0.338
11–15	3.341	1.239	9.009	0.017	2.699	0.835	8.723	0.097
0–10	4.106	1.445	11.664	0.008	3.558	1.252	10.111	0.017

Note: P-values are calculated by conducting logistic regression analysis

**Table 8:** Relationship between the number of seasonal events and children’s prosocial problems

No. events	Crude model				Adjusted model			
	Odds	95% CI		*P*	Odds	95% CI		*P*
≥ 21	Ref				Ref			
16–20	1.731	0.958	3.130	0.069	1.738	0.942	3.206	0.077
11–15	2.413	1.237	4.706	0.010	2.105	1.015	4.366	0.045
0–10	2.962	1.652	5.312	< 0.001	3.184	1.726	5.876	< 0.001

Note: P-values are calculated by conducting logistic regression analysis

## Discussion

This study aimed to quantify the relationship between the number of family events attended and children’s problematic and prosocial behaviors. Children from families who participated in fewer family events tended to have higher problematic behavior risk scores and lower prosociality scores. Conversely, children from families who participated in more family events tended to have lower problem behavior risk scores and higher prosociality scores. This suggests that children’s social adaptation skills improve through participation in family events.

Family events are a particularly important part of a child’s social development. As such events generally require the involvement of the whole family, they facilitate cooperation and empathy development in children [3]. Examples include family Christmas preparations, festivities, and birthday celebrations. Through collaboration with their family members, children learn to understand the division of roles, planning, and the implementation of plans. Family events can also help children develop their communication skills as they provide them with opportunities to communicate with their family members [1].

Through conversations, discussions, and the exchange of ideas, children can express themselves and gain a deeper understanding of others. Family events also provide opportunities to share appreciation and memories, allowing children to learn how to express emotions and gratitude. Moreover, family events provide opportunities to share traditions and culture, through which children can understand their roots and cultural backgrounds and develop their identities. For example, participating in certain religious or ethnic celebrations can help children recognize their status and beliefs, as well as appreciate their similarities and differences from others. Furthermore, family events provide an opportunity to strengthen family bonds, fostering a sense of stability and self-worth, as well as a sense of family bonding and love.

For children to develop social competence, they should have diverse experiences [17, 18]. Exposure to people from different traditions, cultures, and backgrounds helps children understand different perspectives and values, develop flexible thinking, and become more empathetic. For example, attending an international school or growing up in a multicultural neighborhood exposes children to different languages, customs, religions, and foods. Exposure to art, music, sports, and natural sciences can help children develop their interests and talents, as well as improve their creativity and problem-solving skills. Out-of-school learning activities, such as hands-on experiences and field trips, are also important.

Finally, family events provide opportunities to share family history and stories, which are important for identity formation.

### Limitations

This study has some limitations. First, as it was a cross-sectional study, a causal relationship could not be established. Thus, longitudinal research should be conducted in the future to explore changes over time. Second, the questionnaires were completed by children and their mothers. Including other respondents (e.g., teachers) would broaden our understanding of this topic. Third, as indicated in Table 4, no significant difference was observed in the association between a family’s socioeconomic status and the number of family events. However, economic factors may influence the quality and quantity of family events, especially in low-income families. Thus, future research should evaluate the quality and quantity of family events taking economic factors into account. Finally, this study did not explore annual events with non-family members. These events may be relevant as they allow children to interact with their peers and people in their community and can help them increase their awareness of life outside their homes [19]. Thus, future research should verify the effectiveness of events held outside the home (e.g., at school or preschool). If such events prove to be effective, they could support children in unstable home environments.

## Conclusions

Our results suggest that a greater number of family events may reduce the risk of behavioral problems in children and increase prosociality. They further suggest that children’s social adaptation skills improve through participation in events with their families. Therefore, family events are important as they may have a positive effect on children’s development.

## Electronic supplementary material

Below is the link to the electronic supplementary material.

Supplementary Material 1

## Data Availability

The datasets generated and/or analyzed during the current study are available from the corresponding author upon reasonable request.

## Abbreviations

ANOVA: Analysis of variance
SDQ: Strengths and Difficulties Questionnaire
