# Supplementary material for: Family events and child behavior in late childhood: a cross-sectional study
Source: BMC Pediatr. 2024 Nov 20;24:754. doi: 10.1186/s12887-024-05233-9 (PMC11577916; doi:10.1186/s12887-024-05233-9)
Supplement: Supplementary file 1 — Supplementary Material 1 [file 12887_2024_5233_MOESM1_ESM.docx]

**Supplementary Table 1** Relationship between the number of seasonal events and children’s problem behaviors

|  | Crude model | | | | | Adjusted model | | | | |
| --- | --- | --- | --- | --- | --- | --- | --- | --- | --- | --- |
|  | *B* | *SE (B)* | *β* | *P* | *Adjusted R^2^* | *B* | *SE (B)* | *β* | *P* | *Adjusted R^2^* |
| Number of seasonal events | -.088 | .031 | -.113 | .004 | .013 | -.065 | .032 | -.082 | .045 | .038 |

**Supplementary Table 2** Relationship between the number of seasonal events and children’s prosocial behaviors

|  | Crude model | | | | | Adjusted model | | | | |
| --- | --- | --- | --- | --- | --- | --- | --- | --- | --- | --- |
|  | *B* | *SE (B)* | *β* | *P* | *Adjusted R^2^* | *B* | *SE (B)* | *β* | *P* | *Adjusted R^2^* |
| Number of seasonal events | .066 | .014 | .187 | <.001 | .035 | .058 | .015 | .160 | <.001 | .056 |
